# Identification of 15 candidate structured noncoding RNA motifs in fungi by comparative genomics

**DOI:** 10.1186/s12864-017-4171-y

**Published:** 2017-10-13

**Authors:** Sanshu Li, Ronald R. Breaker

**Affiliations:** 1 Institute of Genomics, School of Biomedical Sciences, Huaqiao University, 668 Jimei Road, Xiamen, 361021 China; 2 Howard Hughes Medical Institute, Yale University, Box 208103, New Haven, CT 06520-8103 USA; 3 Department of Molecular, Cellular and Developmental Biology, Yale University, Box 208103, New Haven, CT 06520-8103 USA; 4 Department of Molecular Biophysics and Biochemistry, Yale University, Box 208103, New Haven, CT 06520-8103 USA

**Keywords:** group I, ncRNA, ribozyme, self-cleaving, self-splicing, snoRNA, uORF

## Abstract

**Background:**

With the development of rapid and inexpensive DNA sequencing, the genome sequences of more than 100 fungal species have been made available. This dataset provides an excellent resource for comparative genomics analyses, which can be used to discover genetic elements, including noncoding RNAs (ncRNAs). Bioinformatics tools similar to those used to uncover novel ncRNAs in bacteria, likewise, should be useful for searching fungal genomic sequences, and the relative ease of genetic experiments with some model fungal species could facilitate experimental validation studies.

**Results:**

We have adapted a bioinformatics pipeline for discovering bacterial ncRNAs to systematically analyze many fungal genomes. This comparative genomics pipeline integrates information on conserved RNA sequence and structural features with alternative splicing information to reveal fungal RNA motifs that are candidate regulatory domains, or that might have other possible functions. A total of 15 prominent classes of structured ncRNA candidates were identified, including variant HDV self-cleaving ribozyme representatives, atypical snoRNA candidates, and possible structured antisense RNA motifs. Candidate regulatory motifs were also found associated with genes for ribosomal proteins, *S*-adenosylmethionine decarboxylase (SDC), amidase, and HexA protein involved in Woronin body formation. We experimentally confirm that the variant HDV ribozymes undergo rapid self-cleavage, and we demonstrate that the SDC RNA motif reduces the expression of SAM decarboxylase by translational repression. Furthermore, we provide evidence that several other motifs discovered in this study are likely to be functional ncRNA elements.

**Conclusions:**

Systematic screening of fungal genomes using a computational discovery pipeline has revealed the existence of a variety of novel structured ncRNAs. Genome contexts and similarities to known ncRNA motifs provide strong evidence for the biological and biochemical functions of some newly found ncRNA motifs. Although initial examinations of several motifs provide evidence for their likely functions, other motifs will require more in-depth analysis to reveal their functions.

**Electronic supplementary material:**

The online version of this article (10.1186/s12864-017-4171-y) contains supplementary material, which is available to authorized users.

## Background

The fungal kingdom is an abundantly represented division of the eukaryotic domain of life. It has been estimated that over 1.5 million fungal species could exist, although only about 74,000 have been documented [1, 2]. Numerous species of fungi are major contributors to the composition of many ecosystems where they play important roles in the environment ranging from the simple promotion of organic material decomposition to the formation of essential symbiotic relationships with bacteria, plants and animals [3]. Fungi also serve as important sources for certain human foods, medicines and industrial agents. However, fungi can also be pathogens of many important crop plants, agricultural animals, and of humans. Given the wide-ranging importance of fungal species, deeper explorations of their conserved genetic elements are warranted.

The emergence of inexpensive and efficient DNA sequencing methods has facilitated the determination of the genomic DNA sequences of many fungal species. The yeast *Saccharomyces cerevisiae* provided the first genome of a eukaryote to be completely sequenced [4]. This was followed quickly by the sequencing of additional fungal genomes, including *Schizosaccharomyces pombe* [5], and *Neurospora crassa* [6]. *N. crassa* was the first filamentous species to have its genome sequenced, and it is estimated to possess about double the number of genes predicted to be present in the 6,000-gene *S. cerevisiae*. So far more than 100 fungal species have been fully sequenced [7]. These genome sequences serve as excellent resources for conducting comparative genomics analyses to find common features among diverse fungal species.

Of particular interest to us are structured ncRNAs, such as riboswitches [8] and ribozymes [9], which perform gene regulation, RNA processing, or other important biochemical functions. Such ncRNAs are likely to remain hidden in the genomes of organisms because there is no simple strategy known that can be implemented to comprehensively predict their existence. By contrast, many protein-coding genes contain long open reading frames (ORFs) that are easily revealed by computer-assisted search algorithms. Initially, simple clues regarding the existence of ncRNAs were used to identify novel RNA motifs in bacteria. For example, large gaps between protein-coding regions along with high sequence conservation among closely related bacterial species typically are signatures of ncRNAs [10, 11]. Similarly, orphan promoter or terminator sequences not immediately associated with an ORF [10, 12], GC-rich regions in an organism with a high AT genomic sequence composition [13], and conserved RNA secondary structures [14–16] also have been used to identify structured ncRNAs.

A variety of complex approaches and algorithms that exploit these and other characteristics have since been developed to search for and annotate ncRNAs in species from all three domains of life [17, 18]. Some of these algorithms have been successfully applied to identify additional representatives of known ncRNAs and to discover novel classes present in fungal genomes [12, 19–23]. Even from the earliest computer-assisted searches for ncRNAs in fungi conducted in the 1990s, evidence emerged that many such RNAs remained to be discovered. For example, by detecting RNA polymerase III transcripts and analyzing large noncoding gaps present in the *S. cerevisiae* genome, 16 candidate ncRNAs were identified [12]. Soon thereafter, a total of 22 novel small nucleolar RNAs (snoRNAs) that guide RNA methylation were identified from this same genome by using comparative sequence analysis methods [19]. More recently, RNA transcriptomics methods have been used to identify dozens of additional ncRNAs from fungal species such as *Aspergillus fumigatus* [24] and *Trichophyton rubrum* [25]. These findings suggest that additional ncRNA classes remain to be discovered among the many diverse species of fungi.

Each of the previous computational searches noted above largely focused on a single fungal genome, or a few very closely related genomes. However, given the growth in fungal DNA sequence databases, we sought to identify structured ncRNA candidates that are present in a large diversity of fungal species. Most widely-distributed ncRNAs tend to carry only short stretches of conserved sequences interspersed among structural elements that are only poorly conserved at the nucleotide level [26]. As a result, some successful computational search strategies for structured ncRNA candidates use algorithms such as QRNA [15], RNAz [27], or CMfinder [28], which search for nucleotide covariation or other evidence of structure formation and conservation, rather than just searching for conserved sequence. A computational search across numerous fungal species increases the opportunity to observe nucleotide covariations indicative of structure conservation.

In the current study, we used a computational pipeline similar to that used previously to identify numerous structured ncRNAs in bacteria [29–32]. This pipeline relies on CMfinder [28], which uses an expectation maximization algorithm analogous to MEME [33] to identify probable RNA secondary structures from unaligned sequences via covariance models [26, 34]. To improve the accuracy of consensus secondary structure predictions, a thermodynamic model for base-pairing prediction is also included in CMfinder. Our searches were limited to noncoding regions of 2811 fungal genomes (see the databases for species names), including both intergenic regions and intronic regions. We believe this to be the ideal place to search because eukaryotic TPP riboswitches (the only riboswitch class found in fungi and plants) are commonly located in introns where they regulate alternative splicing upon binding this essential coenzyme [8].

Our pipeline revealed the existence of 15 classes of candidate structured ncRNAs (for a complete list of motifs and species, see Additional file 1: Table S1). These motifs include novel HDV ribozyme variants, non-typical snoRNAs, two structured antisense RNA motifs associated with the chromatin remodeling complex, and various other RNA motifs associated with genes coding for SAM decarboxylase, amidase, and Woronin body proteins. We experimentally confirmed that HDV variants are self-cleaving ribozymes that exhibit catalytic characteristics similar to known HDV ribozymes in other organisms. We also confirmed that the SDC motif is a translational repressor that coordinates the expression of a small upstream open reading frame (uORF) to regulate SAM decarboxylase biosynthesis. Reverse transcription and polymerase chain reaction (RT-PCR) analyses of intronic examples reveal that they likely are involved in the regulation of alternative splicing. These findings demonstrate that numerous well-conserved structured RNA elements are used by a diverse collection of fungi to serve important biochemical roles. Furthermore, this ncRNA discovery pipeline could be used to search through the genomes of other eukaryotic species to discover additional novel structured ncRNAs.

## Results and discussion

### Identification of candidate structured ncRNAs

Promising ncRNA motifs were identified by initially applying our computational pipeline to fungal genome sequences present in NCBI RefSeq Release 29 [35], and later data from RefSeq Release 62 were incorporated into this study. Refinement of the list of representatives, sequence alignments and conserved sequence/structural models were completed with the sequence database as updated in RefSeq Release 75.

**Fig. 1:**
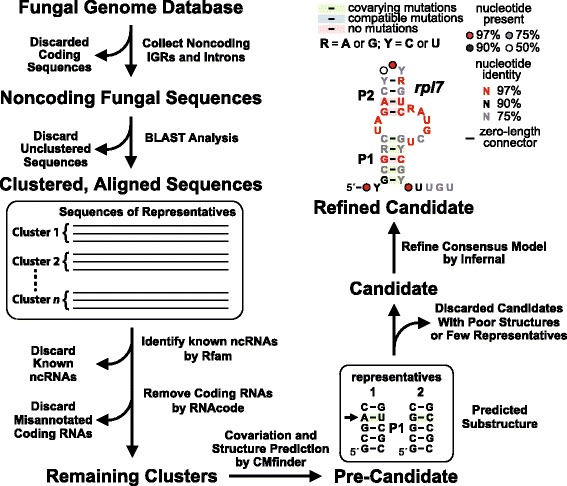
Schematic representation of the workflow for the discovery of novel fungal ncRNA candidates. Noncoding IGRs and intron sequences were analyzed by BLAST to identify clusters of DNA sequences that are similar. The clusters were reduced in number by removing examples that are similar to known RNA motifs present in the Rfam database, and by searching for unannotated protein coding regions by using RNAcode [38]. The remaining clusters were examined by using CMfinder [28] to seek evidence for nucleotide sequence covariation and to develop preliminary secondary structure models. Pre-candidate RNA motifs (P1 designates pairing element 1; arrow identifies covariation site) with only a few covariations and few representatives were discarded. Finally, Infernal was used to search for additional representatives that might have been missed in the initial search, and to refine the consensus sequence and secondary structure model. The refined candidate ncRNA depicted is the novel motif *rpl7* reported in this study

Briefly, the workflow for discovering structured ncRNAs (Fig. 1) involved extracting noncoding fungal DNA sequences as informed by pre-existing genome annotations, clustering of similar sequence regions by using BLAST [36], and filtering to remove clusters matching known ncRNAs present in Rfam [37] or to remove those with extensive protein-coding potential by using RNACode [38]. This process yielded many “pre-candidate” motifs that required further analysis to assess their relative likelihoods of functioning as common fungal ncRNAs. Each pre-candidate was subjected to analysis by CMfinder, which in part uncovers evidence for nucleotide sequence covariation that is indicative of secondary structure formation. If nucleotides in a predicted base-paired stem frequently co-vary in a manner that suggests conservation of the stem, then the cluster of RNAs was considered a strong candidate for having ncRNA function. Iterative analysis of conserved sequences and substructures, augmented by the discovery of additional representatives, was conducted using Infernal [39] to yield a refined sequence and structure model for the ncRNA candidate.

### Analysis of candidate structured ncRNAs

Each ncRNA candidate was evaluated based on the complexity of its conserved sequence and structure model, its common genomic locations, and its phylogenetic distribution, among other features. Below are described the most promising candidate motifs that likely serve as functional ncRNAs. The alignments of these high-ranking motifs in Stockholm format are presented as supplemental material (Additional files 2, 3, 4, 5, 6, 7, 8, 9, 10, 11, 12, 13, 14, 15 and 16).

#### HDV self-cleaving ribozyme variants

**Fig. 2:**
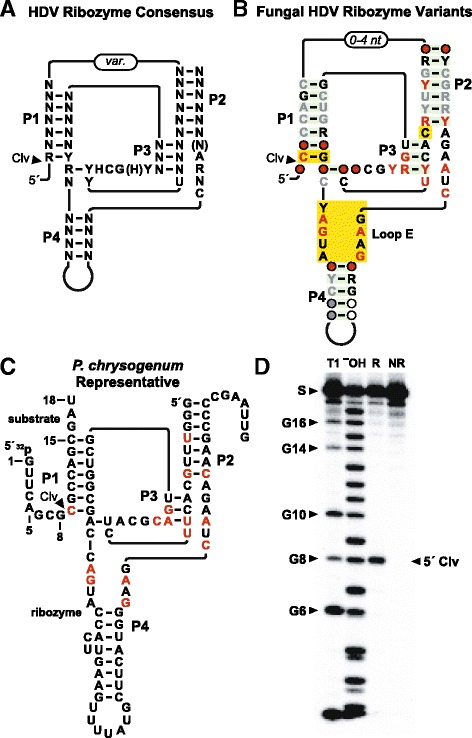
HDV ribozyme consensus models and the characteristics of a newly found HDV ribozyme representative from fungi. **a** General consensus for HDV self-cleaving ribozymes as reported previously [49]. N designates any nucleotide, H designates adenosine, cytidine or uridine, and parentheses identify optional nucleotides. Solid lines indicate zero-length connectors with the exception of a variable-length connector labeled *var*. The arrowhead identifies the site of cleavage (Clv). **b** Consensus sequence and secondary structure model for HDV ribozyme variants identified in fungi. Yellow boxes encompass nucleotides and structures that are different from the general consensus depicted in a, including a “loop E” motif. Other annotations are as described for Fig. 1. **c** Sequence and predicted secondary structure of a bimolecular ribozyme construct derived from an HDV ribozyme from the fungus *P. chrysogenum*. An 18-nucleotide “substrate” strand was separated from a larger “ribozyme” strand by disconnecting the two sub-domains at the junction between P1 and P2. Nucleotides depicted in red match the highly-conserved positions in the fungal HDV ribozyme consensus in Fig. 2b. **d** PAGE separation of 5ˊ ^32^P-labeled substrate RNA after partial digestion with RNase T1 (T1), partial digestion with alkali (^–^OH), or incubation with 100 nM of the ribozyme strand (R) of the *P. chrysogenum* bimolecular construct under permissive reaction conditions (See Methods). NR designates no reaction. Bands corresponding to the substrate (S), 5ˊ cleavage product (5ˊ Clv) and various products generated by RNase T1 cleavage after G residues are also identified

The first member of the HDV ribozyme class [40, 41] to be reported was identified in the antigenomic sequence of the Hepatitis Delta Virus [42]. Since this initial HDV ribozyme discovery, numerous self-cleaving RNAs that conform to this same general consensus sequence and secondary structure model have been discovered [43–45]. Despite the relatively large size of this ribozyme, there are only a few highly conserved nucleotides interspersed among the four extended base-paired regions that form its unique nested pseudoknot architecture [46] (Fig. 2a). The vast majority of HDV ribozyme representatives have been discovered by using a computational strategy that only seeks RNAs conforming to this unique secondary structure, while largely ignoring the few conserved nucleotides [44, 45]. Thus, bioinformatics search methods that also take into consideration conserved nucleotides have the potential to uncover additional representatives.

Our computational pipeline has uncovered a total of 230 representatives of a motif with considerable similarity in sequence and secondary structure to the known members of the HDV self-cleaving ribozyme class (Fig. 2b). These RNAs are present among 26 fungal species. The most noteworthy differences between these new RNAs and the previously published consensus (Fig. 2a) are (i) a C-G base-pair typically represents the otherwise more general R-Y base-pair at the ribozyme cleavage site, (ii) the P4 stem is partially replaced by an E loop [47–49] RNA motif, and (iii) an additional nucleotide (most commonly a C residue) is inserted between the P2 and P3 stems. The latter two differences violate the criteria used previously [41, 44, 45] to find additional members of the HDV ribozyme class, which in part explains why these distinctive fungal examples remained undiscovered. Only 11 fungal HDV ribozyme examples that conform to the published consensus had been discovered previously, suggesting that members of the unusual variant type revealed by our search method predominate in fungi.

Given the distinctive features of the newly-found fungal representatives of this motif, and given their widely variable gene associations, we chose to determine whether members can cleave RNA. Bimolecular substrate-ribozyme complexes were constructed for two examples derived from different organisms. For example, a bimolecular construct based on a representative from *Penicillium chrysogenum* (Fig. 2c) promotes cleavage of the RNA substrate strand at the base of the P1 stem (Fig. 2d). This cleavage site precisely matches that expected for HDV ribozymes, based on the similarity between the predicted structure of the fungal RNAs and the previously-established architecture of self-cleaving ribozymes belonging to the HDV class [46].

**Fig. 3:**
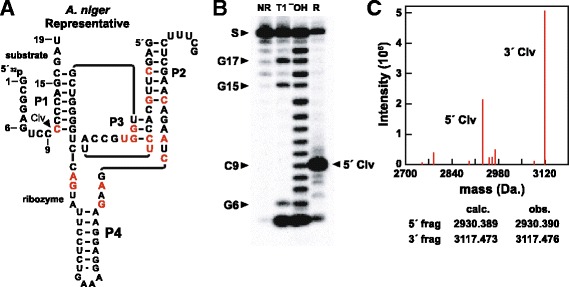
Sequence, structure and activity of the bimolecular HDV construct from *Aspergillus niger.* **a** Sequence and secondary structure model for an HDV variant ribozyme from *A. niger*. Annotations are as described for Fig. 2c. **b** PAGE separation of 5ˊ ^32^P-labeled substrate RNA after partial digestion with RNase T1 (T1), partial digestion with alkali (^–^OH), or incubation with 100 nM of the ribozyme strand (R) of the *A. niger* bimolecular construct under permissive reaction conditions (See Materials and methods). NR designates no reaction. Bands corresponding to the substrate (S), 5ˊ cleavage product (5ˊ Clv) and various products generated by RNase T1 cleavage after G residues are also identified. **c** Mass spectrum analysis of the cleavage products. Peaks that are close to calculated masses of the 9-nucleotide 5´ Clv and 10-nucleotide 3´ Clv products are noted on the graph. The calculated (calc.) and observed (obs.) masses for the cleavage products are listed

Similar results were observed for another bimolecular RNA construct derived from the fungal species *Aspergillus niger* (Fig. 3a). Again, the cleavage site for this fungal HDV ribozyme variant corresponds to that expected for more conventional HDV ribozymes as determined by gel mobility (Fig. 3b) and analysis by mass spectrometry (Fig. 3c) of the substrate cleavage products. Moreover, the masses of the products are consistent with a ribozyme mechanism wherein a 2ˊ-oxygen atom serves as a nucleophile to attack the adjacent phosphorus center to yield cleavage products with 2ˊ, 3ˊ-cyclic phosphate and 5ˊ hydroxyl termini on the 5ˊ and 3ˊ cleavage fragments, respectively. This is the same mechanism used by all other small self-cleaving ribozymes discovered to date [50].

#### SDC motif

**Fig. 4:**
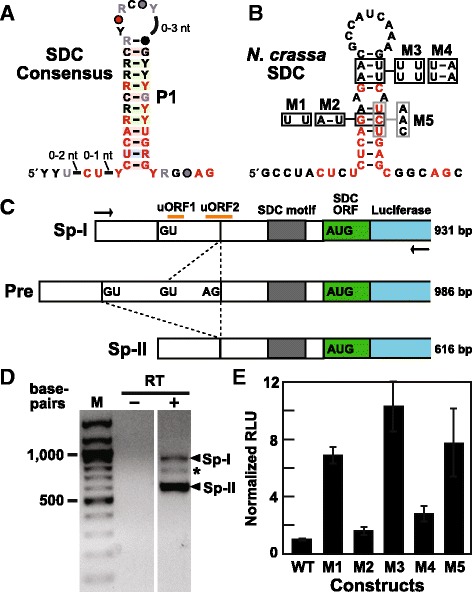
Structure and gene control function of the SDC motif. **a** Consensus model depicting the conserved sequences and predicted secondary structure of SDC motif RNAs. Annotations are as described in Fig. 1. **b** Sequence and secondary structure model for the SDC motif representative from *N. crassa*. Nucleotides depicted in red correspond to the most highly conserved nucleotides present in the consensus sequence in a. M1 through M5 identify nucleotide differences at the positions indicated in mutant constructs used to assess the importance of the P1 stem to gene expression. **c** Schematic representation of the genetic elements present near the SDC motif, including the location of the luciferase reporter gene used for RT-PCR and reporter-fusion gene expression assays. Arrows identify primer binding sites used for RT-PCR. Dashed lines identify splicing variations using one of the two 5ˊ splice sites (GU) and the 3ˊ splice site (AG) that can convert the precursor mRNA (Pre) into the alternative splicing products Sp-I and Sp-II. The graphic is not drawn to scale. **d** Agarose gel separation of RT-PCR products generated from SDC reporter fusion transcript in *N. crassa*. The absence (–) or presence (+) of reverse transcriptase (RT) in the assay is indicated. The asterisk denotes an RT-PCR product whose identity was not confirmed by DNA sequencing. M indicates double-stranded DNA markers. The two images depict neighboring parts of the same gel. **e** Gene expression of wild-type (WT) and mutant SDC reporter-fusion constructs. Relative light units were normalized to WT (value of 1). The values are an average of three independent replicates, and error bars represent standard deviation

The SDC motif, represented by 34 distinct examples from 26 fungal species, typically forms a small hairpin with an 11-base-pair stem (Fig. 4a). However, SDC motifs can exhibit some variation in stem integrity and length (e.g. see Fig. 4b for the representative from *N. crassa*). Each representative is located immediately upstream of a SAM (*S*-adenosylmethionine) decarboxylase gene, suggesting that hairpin formation and the associated conserved nucleotides are important for regulation of the SDC gene. In *N. crassa*, there appear to be two uORFs (Fig. 4c), which are short peptide-coding regions located in some mRNAs a short distance upstream of a main open reading frame. Such uORF regions are commonly involved in controlling translation initiation of the adjoining gene [51, 52]. For example, in *N. crassa*, high arginine concentrations cause ribosomes to increase stalling within the *arg-2* uORF, which reduces translation initiation at the main ORF located immediately downstream [53, 54]. Both arginine and newly synthesized uORF-derived peptides are required for ribosomal stalling.

The SDC enzyme catalyzes the synthesis of *S*-adenosylmethionine to provide propylamine for the production of polyamines, such as spermidine and spermine [55, 56]. The production of polyamines is known to be highly regulated by cells at multiple levels including transcription, translation, enzyme activation and protein degradation. At the translational level, mammalian and plant SDC expression is primarily regulated by the expression of uORFs located in their 5´ UTRs [55, 56], and to a lesser extent by secondary structures formed by the 5ˊ UTR [57]. Indeed, polyamine-responsive gene control very commonly involves uORF elements [58].

The two uORFs associated with the SDC motif of *N*. *crassa* are located nearby two consensus 5ˊ splice sites and one 3ˊ splice site (Fig. 4c). Given that some TPP riboswitches in fungi control both alternative splicing and uORF expression [55], we initially speculated that the SDC motif might be part of a complex set of regulatory elements that similarly controls SDC gene expression. To examine the biological function of the SDC motif, the 5′ UTR of the *N. crassa* SDC gene including a portion of the SDC ORF was fused in-frame with a luciferase reporter gene. This construct (Pre, Fig. 4c) was transformed into *N. crassa* cells and the presence of various RNA transcripts was examined by RT-PCR (Fig. 4d). The mRNA precursor was found to be efficiently alternatively spliced into two predominant forms called Sp-I, which contains two predicted uORFs, and Sp-II, which lacks these two uORFs (though the 3ˊ half of uORF 2 is still present) (Fig 4c, d). Therefore, any effects on expression of the SDC ORF possibly caused by the uORFs cannot occur with the Sp-II form of the processed mRNA.

We then assessed the importance of the SDC motif hairpin to gene expression by examining luciferase activity in fungal preparations carrying the WT and M1 through M4 reporter-fusion constructs. Disruption of the P1 stem by changing one (M1), two (M3), or three (M5) base-pairs to mismatches causes a substantial increase in reporter gene expression relative to that observed with the WT construct (Fig. 4e). By introducing additional mutations that restore base-pairing (M2, M4), these mutant constructs exhibit gene expression levels that approach that of the WT construct. These findings indicate that the SDC motif behaves as a negative regulatory element to inhibit SDC gene expression.

RT-PCR analysis on the variant constructs revealed that the disruptive SDC motif mutations M1 and M3 do not influence the levels of Sp-I and Sp-II mRNA products (data not shown), suggesting that the SDC motif regulates gene expression in a manner that does not directly involve alternative splicing. Moreover, we did not observe evidence for specific binding of spermine or spermidine to a representative of the RNA motif by using in-line probing [59, 60] assays (data not shown). Therefore, the mechanism of regulation by the SDC motif appears to differ from that observed for some fungal TPP riboswitches that regulate alternative splicing and the translation of uORFs [61]. Additional studies will be necessary to determine (i) if alternative splicing of the SDC precursor RNA is regulated, (ii) if uORF-mediated regulation of SDC gene expression occurs, (iii) how the SDC motif hairpin suppresses gene expression, and (iv) how SDC motif structure can be naturally manipulated to affect expression.

#### *amd* motif

**Fig. 5:**
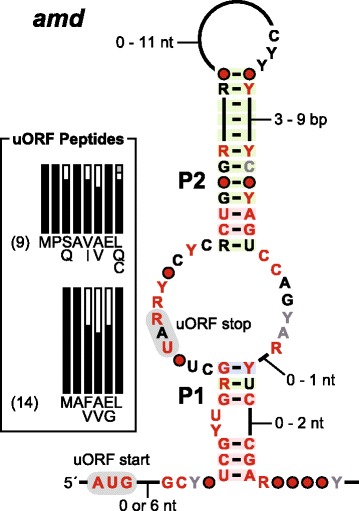
Consensus sequence, structure, and putative uORF peptide products for the *amd* motif. RNA annotations are as described for Fig. 1. Plots for the peptide sequences are proportional to the amino acids denoted, where black, white and gray bars indicate the most- to least-common amino acid, in the order presented. Numbers in parentheses indicate the numbers of representatives predicted to code for 8- and 6-amino acid peptides

A total of 23 unique examples of the *amd* motif (Fig. 5) have been identified among 20 fungal species. The consensus sequence and secondary structure model based on these sequences reveal that the motif likely adopts an elongated two-stem junction wherein the most-highly conserved nucleotides reside in the internal loop and in sections of P1 and P2 nearest to this loop. Moreover, the nucleotides immediately upstream of P1, along with several others extending into the internal loop, appear to code for a short uORF. This indicates that the ORF and overlapping RNA structure of the *amd* motif might collaboratively regulate the downstream gene. Of the 23 unique *amd* motif examples, nine members carry a uORF that codes for eight amino acids, and 14 members carry a uORF that codes for only six amino acids (Fig. 5). The putative peptides are highly conserved, and the last five amino acids of both groups carry the sequence A(V/F)(A/V)EL.

Each *amd* motif example resides upstream of a protein-coding gene of unknown function, making it difficult to formulate hypotheses regarding the function and mechanism of this putative ncRNA element and associated uORF. In more than nine species, the associated gene is annotated as coding for an amidase enzyme, whose function is to hydrolyze amide functional groups to yield ammonia and a carboxylic acid group. Given the presence of a glutamate codon in the penultimate position of the uORF, the amidase activity might be related to the production of this amino acid. For example, a shortage of glutamine might cause the uORF system to trigger expression of a glutaminase ORF to convert glutamine to glutamate. A previous attempt to create an *amd* gene (*NCU05182*) knockout in *N. crassa* was unsuccessful [62]. The heterokaryotic strain, but not the homokaryotic strain, survived, indicating that the gene associated with the *amd* motif is critical for cell survival. Given this experimental complication, we did not pursue additional validation studies.

#### *ies6* motif

**Fig. 6:**
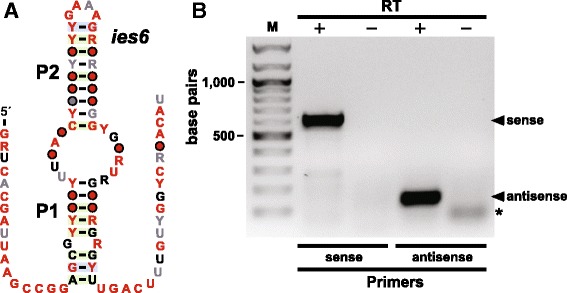
The *ies6* RNA motif and its expression. **a** Consensus sequence and secondary structure of the *ies6* motif. Annotations are as described for Fig. 1. **b** Agarose gel separation of RT-PCR products generated by using primers specific for the sense or antisense transcripts as designated. Bands corresponding to the double-stranded DNA products derived from the sense and antisense RNA templates are indicated. M designates double-stranded DNA size markers, and lanes containing PCR products generated with (+) or without (–) the use of reverse transcriptase (RT) are identified. The asterisk identifies a spurious PCR product

The *ies6* motif (Fig. 6a) includes 34 examples from 20 species of fungi. This RNA motif forms an extended hairpin structure with one small and one large internal loop. The hairpin loop conforms to a GNRA tetraloop sequence [63], which is frequently found in structured RNAs. GNRA tetraloops can form tertiary interactions with tetraloop receptor structures [64, 65]. This fact, coupled with the extensive conservation on each side of the hairpin structure suggests that the consensus motif as presented might represent only a portion of a more complex RNA architecture.

These predicted RNA structures are almost always located adjacent to a gene coding for a protein similar to the chromatin remodeling complex subunit Ies6 (or Ino80 Subunit 6), which is involved in chromatin modification, chromosome segregation and the regulation of telomere length [66–68]. However, the polarity of the RNA motif as depicted (Fig. 6a) is opposite to that of the *ies6* mRNA, because this configuration retains a GNRA tetraloop commonly found in structured RNAs. Therefore we speculated that this motif is likely present in an antisense RNA produced from the same genomic location as the *ies6* gene. To determine if both sense and antisense RNAs corresponding to the *ies6* motif were produced by *N. crassa* cells, we conducted RT-PCR assays. As expected, RT-PCR product bands corresponding to both the sense and antisense RNA transcripts were observed by gel electrophoresis (Fig. 6b).

Antisense transcripts are found in many species where they often participate in the gene regulation. They are able to base-pair to their targets to regulate gene expression through various mechanisms including transcription attenuation, translation inhibition, primer maturation inhibition, splicing regulation, nuclear retention, and mRNA degradation and stabilization [69–72]. However, additional experiments will be required to determine if the *ies6* motif is indeed part of an antisense RNA transcript, and what role this motif might play in regulation of antisense production and action.

#### *hexA* motif

**Fig. 7:**
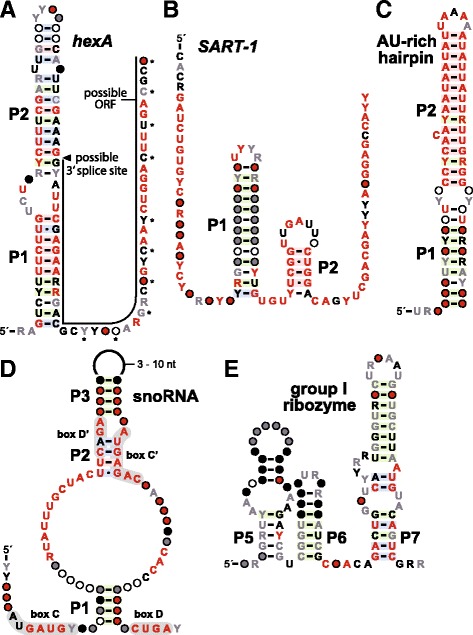
Consensus sequences and secondary structure models for the predicted RNA motifs **a** *hexA*, **b** *SART-1*, **c** AU-rich hairpin, **d** variant snoRNAs, and **e** group I ribozyme region. Annotations are as described for Fig. 1

The *hexA* motif (Fig. 7a) includes 19 examples from 10 fungal species. The predicted secondary structure includes a long hairpin interrupted by two small internal loops. In addition, a long region with sequence conservation lacking evidence of structure extends downstream. The motif is found in the putative 5´ UTRs or introns of genes annotated as encoding either hypothetical proteins (40%) or Woronin body [73, 74] protein Hex subunits (60%). Woronin bodies are fungal-specific organelles that plug the septal pores quickly to prevent a cell from losing its contents during physical damage. Hex subunit proteins are derived from differently spliced mRNA forms and are one of the major and essential components of the Woronin body [74, 75].

Intriguingly, a portion of the sequence forming the right shoulder of the *hexA* motif hairpin closely approximates the consensus for a 3ˊ splice site (Fig. 7a). Moreover, nucleotides within the long 3ˊ tail exhibit evidence that they code for protein. Specifically, the least conserved nucleotides in this region occur at every third position, which might correspond to the wobble position of codons. These characteristics suggest that the *hexA* motif RNA structure might regulate splicing to create an altered ORF sequence for translation. However, at least seven of the representatives of the *hexA* motif are in the opposite direction as the genes encoding Woronin body proteins, whereas at least ten are in the same orientation. This variability in orientation weakens the hypothesis that the motif might be involved in gene regulation by controlling alternative splicing of the adjacent coding region.

#### SART-1 motif

The *SART-1* motif consensus model (Fig. 7b) is based on 12 unique examples from 11 fungal species. The secondary structure can potentially include at least two hairpins, although only P1 is supported by extensive evidence of covariation and the frequent presence of a UNCG tetraloop element. RNA hairpin loops that conform to this consensus are known to be structurally stabilizing to adjoining base-paired regions [76, 77]. Nearly all representatives are located in the same location as the 5´ UTRs of genes that produce a protein of unknown function similar to the mammalian *SART-1* (squamous cell carcinoma antigen recognized by T cells) protein [78]. SART-1 is similar to the yeast Snu66 spliceosomal protein. One *SART-1* RNA representative resides in the location of an annotated intron for the *SART-1* gene. In mammals, the *SART-1* gene encodes split ORFs that are possibly translated by a mechanism of -1 frameshifting.

Importantly, the *SART-1* RNA motif is predicted to be formed by antisense transcripts of the associated gene. This hypothesis is supported by the fact that numerous G-U wobble pairs in the consensus model would otherwise become A-C mismatches if the RNA structure were formed by the sense transcript, which is unlikely. Furthermore, the commonly-occurring UNCG tetraloop structure in P1 would become a CGNA tetraloop in the sense direction, which is not known to confer the same structural benefit as a UNCG tetraloop.

#### AU-rich hairpin motif

Only 12 examples of the AU-rich hairpin motif (Fig. 7c) were identified from three fungal species, and eight of these representatives were from a single species, *Rhizoctonia solani*. Interestingly, two additional examples with considerable sequence and structural similarity were found in the bacterial genome of *Orientia tsutsugamushi*. These two bacterial examples were originally annotated as members of the *mraW* class of putative ncRNAs as reported previously [31]. However, only a small portion of the consensus sequence for *mraW* motif RNAs is similar to the consensus sequence derived from the fungal examples of the AU-rich hairpin motif. Therefore it is not certain whether these similarities are biologically relevant or coincidental.

The function of the bacterial *mraW* motif RNAs remains unknown, and unfortunately the gene associations for the fungal examples are highly variable. As a result there are no compelling clues regarding the possible biological roles of the fungal motif. If additional examples are found in the future, perhaps genomic location data might provide the insight necessary to better formulate testable hypotheses.

#### Atypical snoRNA motif

The atypical snoRNA motif (Fig. 7d) is represented by 81 examples from 52 fungal species. Most of the representatives are located in introns, with the exception of seven that appear to reside apart from introns. For example, in the yeast *Schizosaccharomyces pombe* (NC_003424.3), the motif resides in the intron of the cpc2 gene from nucleotides 2440331 to 2440419. In most instances, this RNA motif is associated with genes encoding guanine nucleotide-binding proteins (G proteins), and more rarely are located near several genes for proteins of unknown function or for sterol 24-C-methyltransferase. G proteins are important signal transduction components whose functions are well established in a diversity of signaling pathways [79, 80], although the functions of the specific G proteins associated with this ncRNA motif are unknown.

These fungal ncRNA candidates exhibit considerable similarity to certain snoRNAs, which guide chemical modifications on other RNAs, including ribosomal RNAs (rRNAs), transfer RNA (tRNAs), and small nuclear RNAs (snRNAs) [81]. One type, called box C/D snoRNAs, have a conserved C box (RUGAUGA) and D box (CUGA). Additional conserved regions called the Cˊ box and Dˊ box mimic the sequence of the C box and D box, respectively. The atypical snoRNA motif examples we identified carry two regions that closely approximate the C box (AUGAUGY) and D box (CUGA), although the apparent Cˊ box (AUGAGAC) and Dˊ box (CAGA) consensus sequences correspond to the consensus snoRNA sequences more poorly.

RT-PCR was used to evaluate the production of the *A. nidulans* representative of this RNA class. Only the RT-PCR product corresponding to the spliced RNA was observed (data not shown), indicating that the intron carrying the atypical snoRNA is efficiently removed from the original transcript. This result might indicate that the intron is always removed, rather than undergoing regulation by the structured element in the intron. If true, then this atypical snoRNA might have a function similar to other snoRNAs. Consistent with this hypothesis is the fact that the highly-conserved nucleotides in the guide sequence region of the atypical snoRNA are complementary to regions within 5.8S rRNA, 18S rRNA, and 28S rRNA, suggesting that members of this ncRNA class direct modifications to these rRNAs.

#### Group I ribozymes

Bioinformatics searches that are guided by consensus sequences and structure models for known RNA classes are likely to miss many representatives, particularly those that can vary considerably from the consensus model. Searches that can uncover conserved sequences and structures without relying on pre-existing consensus models can reveal distal variants of known ncRNA classes. As noted above, our bioinformatics search strategy already has revealed numerous additional representatives of the HDV class of self-cleaving ribozymes (Fig. 2) and members of an atypical snoRNA (Fig. 7d). Similarly, we have identified 208 examples of what appear to be previously unannotated group I self-splicing ribozymes [82] that are present in 114 fungal species (Fig. 7e).

Numerous additional examples of this motif were uncovered in bacteria by searching for sequences conforming to the resulting consensus model based on these fungal representatives (unpublished observations). Most of these newly-found representatives carry readily recognizable structural elements of group I ribozymes, including stems P3 through P7, and the conserved guanosine binding site. Since there is considerable sequence and structural variability at both the 5ˊ and 3ˊ termini of group I ribozymes, we did not further examine each representative to determine if they also carry stems P1, P2, P9, and P10 that are typical of this ribozyme class. However, these RNAs are most likely group I ribozymes that have previously escaped annotation in the genomes we analyzed.

#### *rps0* motif

**Fig. 8:**
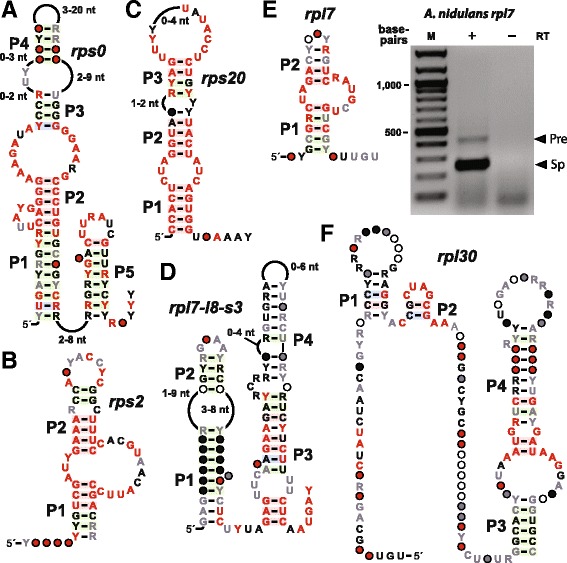
Consensus sequences, secondary structure models, and additional data for RNA motifs associated with ribosomal protein genes. The RNA motifs **a** *rps0*, **b** *rps2*, **c** *rps20*, **d** *rpl7-l8-s3*, **e** *rpl7*, and **f** *rpl30* are named after the associated ribosomal protein genes. The agarose gel image in e reports the results of RT-PCR analysis of splicing products for intronic motif *rpl7* from *A. nidulans*. RT-PCR products derived from unspliced pre-mRNA (Pre, 437 base-pairs) and spliced mRNA (Sp, 253 base-pairs) are identified. M is a DNA marker with 100 base-pair band increments, and RT designates reverse transcription. Other annotations are as described for Fig. 1

We identified many distinct types of candidate structured RNA domains in close association with fungal genes coding for ribosomal proteins. The *rps0* motif is one such representative (Fig. 8a) that included 41 representatives from 25 fungal species. Ribosomal protein genes commonly use a feedback auto-regulation mechanism to regulate their expression in bacteria [83–85]. These systems involve the binding of the ribosomal protein to a special RNA structure commonly located in the 5ˊ UTR of its corresponding mRNA. For example, *E. coli* uses at least 12 distinct RNA structures to regulate the expression of numerous ribosomal proteins [86]. This general mechanism is also used by some eukaryotic species for regulating pre-mRNA splicing [87] or translation [88]. In *S. cerevisiae*, ribosomal protein L32 (RPL32) binds to a structured RNA formed by intronic and exonic sequences within its own mRNA to cause alternative splicing. Moreover, the spliced product can also fold into a very similar structure that still binds the RPL32 protein and inhibits translation [89, 90].

The *rps0* motif identified in the current study consists of two hairpins, one of which carries two conserved 5´-GGGGAAAG sequence elements partly located on each side of an internal loop. All the representatives of this motif are located in the 3´ UTRs of genes encoding 40S ribosomal protein subunit S0 (RPS0). RPS0 proteins are required for the processing of the precursor of 18S rRNAs and the formation of active 40S ribosomal subunits [91]. Given the apparent symmetry of the *rps0* RNA sequence and its location adjacent to the *rpo0* gene, it seems possible that the motif might bind two or more RPS0 proteins to regulate expression of this ribosomal protein factor.

#### *rps2* motif

The *rps2* motif (Fig. 8b) is represented by 13 examples from nine fungal species belonging to four subclasses including *Pichia*, *Candida*, *Lodderomyces* and *Clavispora*. All examples are located in the 5′ UTRs of the genes encoding 40S ribosomal protein S2. Although there are few examples, there are four predicted base-pairs distributed between P1 and P2 that covary in a manner consistent with the predicted secondary structure.

#### *rps20* motif

There are 20 representatives of the *rps*20 motif (Fig. 8c) derived from 13 species distributed among the *Saccharomyces*, *Candida*, *Lachancea*, *Kazachstania* and *Tetrapisispora* genera. All the representatives are located in the 3′ UTRs of the genes encoding 40S ribosomal protein S20. Again, given the limited number and distribution of members of this motif, we observe only two base-pairs with evidence of covariation in the proposed P3 stem. The other two predicted base-paired regions are formed by highly-conserved sequences and therefore further evidence of their formation is currently lacking.

#### *rpl7-l8-s3* motif

The *rpl7-l8-s3* motif (Fig. 8d) forms two long hairpins with internal loops. A total of 29 representatives from 25 species are located mostly in the introns of genes encoding 60S ribosomal proteins L7 and L8, as well as the 40S ribosomal protein S3. L7 and L8 proteins are required for the processing of 60S pre-rRNA, which includes the peptide bond formation center of 28S rRNA. The motif includes a well-conserved purine-rich region located between two internal loops of P2, which is similar to a previously reported ribosomal protein binding site [92]. However, this previously reported motif in *S. cerevisiae*, which is located in the pre-mRNA for ribosomal protein L32, is proposed to be bound by the L32 protein as an unpaired bulge. In contrast, the conserved purine-rich region of the *rpl7-l8-s3* motif is predicted to be part of an extended base-paired structure that includes evidence for some nucleotide covariation, implying that this region might not exist as unpaired RNA.

#### *rpl7* motif

The *rpl7* motif (Fig. 8e) is represented by 22 examples from 17 species. It is a small motif of approximately 30 nucleotides containing some conserved nucleotides in and around a central loop. All examples of this RNA motif are located in the introns of 60S ribosomal protein L7 mRNAs. In the absence of L7, other ribosomal proteins such as L6, L14, L20, and L33 are greatly diminished [93], and so this motif might be important for the coordination of ribosomal protein production.

We also used RT-PCR to test whether splicing occurs. If so, the motif might participate in regulating splicing events, as has been observed for an RNA structure associated with *S. cerevisiae* ribosomal protein L32 (RPL32) [90]. RT-PCR analysis (Fig. 8e) reveals that the L7 pre-mRNA that contains the *rpl7* motif produces at least one major splicing product. This result, along with the location of the *rpl7* motif in introns, is consistent with the hypothesis that the *rpl7* motif might regulate pre-mRNA splicing, perhaps by directly binding to the L7 ribosomal protein.

#### *rpl30* motif

The *rpl30* motif (Fig. 8f) is represented by 47 examples from 26 species. This motif is comprised of a long 5ˊ region with little evidence for structure formation, followed by a region that appears to form a large hairpin structure with a well-conserved purine-rich internal loop. Most representatives are located in the 5´ UTRs of genes encoding 60S ribosomal protein subunit L30. Notably, expression of *S. cerevisiae* ribosomal protein L32 is autogenously regulated by a mechanism wherein the protein binds to a purine-rich internal loop [92]. This precedence strengthens the working hypothesis that the *rpl30* motif is a regulatory RNA structure that serves as a binding partner for ribosomal protein L30 for expression autoregulation.

## Conclusions

Comparative genomics is a powerful approach to evaluate the degree of conservation between even distantly-related genomic DNA sequences and has been widely applied to compare genes that encode proteins [94]. We have used such approaches to discover numerous novel RNA motifs in bacteria [29–32]. However, using comparative genomics to discover structured ncRNAs in eukaryotes has not been commonly pursued, particularly among evolutionarily distant species [20, 95, 96]. Technical advances in analyzing numerous large genomes, such as improvements in computer processing speeds and more effective computational pipelines [28, 39], have created new opportunities to efficiently discover novel eukaryotic ncRNAs. Another advance is the availability of many sequenced eukaryotic genomes, which overcomes the problems otherwise caused by examining only a few phylogenetically similar eukaryotes.

To identify novel fungal ncRNAs, we first extracted IGRs of fungal genomes using available genome annotations. The elimination of coding regions greatly diminishes the computational challenge, and yet enriches the sequence data searched for candidates because known structured RNAs such as ribozymes and riboswitches almost never overlap with large ORFs. Comparative sequence analyses on this select sequence dataset using our pipeline reveal both conserved sequence and secondary structure features that define each of the novel RNA motifs. The list of representatives of promising ncRNA candidates was further expanded by additional homology searches, which iteratively refine the consensus models and allow for additional representatives to be discovered [29].

Through these efforts, we have identified 15 classes of structured ncRNA motifs in fungal genomes, including variants of HDV self-cleaving ribozymes, non-typical snoRNA candidates, the SDC motif, two antisense structured RNAs (*ies6* and *SART-1*), a motif including a uORF that might regulate a gene that is critical for cell survival, a motif involved in Woronin body formation, and six motifs likely involved in regulating ribosomal protein expression. Although the functions of some motifs have been analyzed by using biochemical or genetic methods, many additional experiments will be needed to precisely define the roles and mechanisms for these ncRNA candidates.

Already, it appears that the functional roles of these ncRNAs are diverse. For example, representatives of the variant HDV ribozymes were found to efficiently self-cleave (Fig. 2) by using the same mechanism for phosphoester transfer as other self-cleaving ribozymes. The SDC motif appears to function as a translation repressor that likely coordinates with a uORF in the same 5´ UTR to regulate SAM decarboxylase synthesis (Fig. 4). It appears that the *amd* motif also encodes a conserved oligopeptide wherein this uORF overlaps with a domain of extensive RNA sequence and structure conservation. Possibly, uORF expression regulates the formation of *amd* motif RNA structure bring about the desired biological effect. Finally, RT-PCR results indicate that the function of the *ies6* motif might be performed as part of an antisense transcript (Fig. 5), and that the *rpl7* motif is present in a pre-mRNA that undergoes splicing (Fig. 8).

These findings demonstrate how the analysis of multiple fungal genomes can be used to reveal the existence of numerous structured ncRNA candidates. Similar approaches could be used to reveal the existence of additional structured ncRNAs in other eukaryotic systems whose biochemical and biological functions are highly diverse.

## Methods

### Identification of candidate RNA motifs

Fungal genome sequences including annotations were initially downloaded from the RefSeq Release 29 obtained from NCBI [35] and The Fungal Genomics group at the Broad Institute (http://www.broadinstitute.org/science/projects/fungal-genome-initiative/current-fgi-sequence-projects). The fungal sequences available as of November 2013 (RefSeq Release 62) were incorporated into this study (see also Additional file 17: Table S2).

The procedure to discover structured ncRNAs is outlined in Fig. 1. Briefly, IGRs from all genomes and introns from each annotated gene were extracted. The sequences also were selected by considering alternative splicing information according to the fungal genome annotations. IGRs and introns were compared among species of fungi by NCBI BLAST (version 2.2.23) [36] using parameters –m8 –W7 –e 1e–5. Similar sequences were automatically grouped by BLAST and a PERL script to form different clusters. Each cluster was filtered using BLAST against the Rfam database to remove any known RNAs with parameters –m8 –W10 –e 1e–10. The resulting new candidate clusters were further filtered by RNAcode [38] to remove clusters whose score is greater than 15, indicating that they are potentially protein coding. The remaining clusters were fed into CMfinder [28] to predict RNA structures based on covarying mutations among RNA sequences. Clusters with little covariation or with very few examples (less than three sequences in a cluster) were discarded. Finally, Infernal [39] was used to search for more homologs according to both sequence and structure similarity and to refine the structure of the RNA if more examples were discovered. During the Infernal search, the information about the RNA motif location and associated upstream and downstream genes were also inspected.

### Assessing the novelty of motifs

To determine whether the predicted structured RNA motifs were reported previously, we took out every sequence of each alignment and compared them to sequences in the Rfam database using default parameters [37]. Novel RNA candidates are those not found in Rfam, not highly homologous to RNAs in Rfam, or highly homologous to any known RNAs in other known databases such as NCBI gene bank databases. Notably, 11 sequences of putative HDV-like ribozymes were reported previously [44] and some of them overlapped with our collection, but the rest of the 223 HDV variant representatives were newly identified.

### Ribozyme self-cleavage assays

Self-cleaving ribozyme assays were conducted using reaction conditions similar to those described previously [32]. Synthetic RNA substrates were purchased (Sigma-Aldrich), whereas the enzyme RNA strands were prepared by in vitro transcription [97]. Substrate RNAs were dephosphorylated, 5´-radiolabeled by using γ-^32^P ATP and T4 polynucleotide kinase (New England Biolabs), and purified by denaturing (8 M urea) 20% polyacrylamide gel electrophoresis (PAGE). 5 nM radiolabeled substrate and 100 nM enzyme RNA strands were combined in a reaction mixture containing 30 mM Tris-HCl (pH 7.5 at 23°C), 100 mM KCl, and 20 mM MgCl_2_, and incubated at 23°C for 30 min. The reaction was stopped by adding an equal volume of stop solution containing 90% formamide, 50 mM EDTA, 0.05% xylene cyanol and 0.05% bromophenol blue. Partial alkaline and RNase T1 digests of RNA samples were prepared for marker lanes T1 and ^–^OH as described previously [32]. The reaction products were separated by denaturing 20% PAGE and imaged/quantitated by a PhosphorImager (GE Healthcare).

### Mass spectrometry analysis of cleavage products

Twenty pmol each of the HDV substrate and enzyme RNAs were incubated as described above in a 20 μL reaction for one hour. The reaction products were assayed by monoisotopic (exact mass) spectrometry (Novatia LLC).

### Plasmids and strains

Plasmid pLL07-2-1 was constructed as described previously [98]. To create an in-frame fusion of the luciferase reporter gene to the *NCU01083* ORF, a DNA fragment of 967 base-pairs (including the first 62 base-pairs of the main ORF) was amplified by primers sdc-F and sdc-R (Additional file 18: Table S3), which include *Eco*RI and *Xba*I restriction sites, respectively. The fragment was sub-cloned into a pCR2.1-TOPO vector (Life Technologies). After confirmation by sequencing (Keck Foundation Biotechnology Resource Center at Yale University), the fragment was digested, purified by agarose gel electrophoresis, and inserted into the plasmid pLL07-2-1 at the *Eco*RI and *Xba*I sites. For mutant constructs, site-directed mutagenesis (Stratagene) and two-step PCR were used to make mutations at the appropriate sites.

### *N. crassa* transformation and luciferase assay

Electroporation transformation into *N. crassa* 87-74 (*bd; frq+ a; his-3*) [99] and luciferase assays were performed as described previously [98].

### Reverse transcriptase polymerase chain reaction (RT-PCR)

RT-PCR was carried out as described previously [98]. Primers that are complementary to exon sequences located immediately upstream or downstream of the intronic motifs were used to detect alternative splicing products. For antisense motif *ies6* two pairs of primers were designed to detect antisense and sense transcripts independently. Sequences of primers and predicted sizes of RT-PCR products are listed in Additional file 18: Table S3. During the RT-PCR process, a negative control, in which the RT reaction lacked reverse transcriptase, was included to ensure there was no DNA contamination.

## Additional files

Additional file 1: Table S1. Discovered ncRNA Information. Properties of and species for each ncRNA discovered. (DOCX 24 kb)

Additional file 2: HDV ribozyme sequences. sto file for HDV ribozyme sequences and their alignment. (STO 40 kb)

Additional file 3: SDC motif sequences. sto file for SDC motif RNAs and their alignment. (STO 3 kb)

Additional file 4: *amd* motif sequences. sto file for *amd* motif sequences and their alignment. (STO 4 kb)

Additional file 5: *ies6* motif sequences. sto file for *ies6* motif sequences and their alignment. (STO 5 kb)

Additional file 6: *hexA* motif sequences. sto file for *hexA* motif sequences and their alignment. (STO 2 kb)

Additional file 7: SART-1 motif sequences. sto file for SART-1 motif sequences and their alignment. (STO 2 kb)

Additional file 8: AU-rich motif sequences. sto file for AU-rich motif sequences and their alignment. (STO 1 kb)

Additional file 9: Atypical snoRNA motif sequences. sto file for atypical snoRNA motif sequences and their alignment. (STO 15 kb)

Additional file 10: Group I ribozyme sequences. sto file for group I ribozyme sequences and their alignment. (STO 59 kb)

Additional file 11: *rps0* motif sequences. sto file for *rps0* motif sequences and their alignment. (STO 9 kb)

Additional file 12: *rps2* motif sequences. sto file for *rps2* motif sequences and their alignment. (STO 1 kb)

Additional file 13: *rps20* motif sequences. sto file for *rps20* motif sequences and their alignment. (STO 2 kb)

Additional file 14: *rps7-l8-s3* motif sequences. sto file for *rps7-l8-s3* motif sequences and their alignment. (STO 5 kb)

Additional file 15: *rpl7* motif sequences. sto file for *rpl7* motif sequences and their alignment. (STO 1 kb)

Additional file 16: *rpl30* motif sequences. sto file for *rpl30* motif sequences and their alignment. (STO 12 kb)

Additional file 17: Table S2. Genome database lists. A listing of the genomic database files used for computational searches. (DOCX 13 kb)

Additional file 18: Table S3. PCR primers. DNA sequences of PCR primers used for the study. (DOCX 17 kb)

## Abbreviations

IGR: Intergenic region ncRNA noncoding RNA ORF Open reading frame PAGE Polyacrylamide gel electrophoresis
RP: Ribosomal protein rRNA ribosomal RNA RT-PCR Reverse transcription – polymerase chain reaction SAM *S*-adenosylmethionine SDC *S*-adenosylmethionine decarboxylase snoRNAs small nucleolar RNAs snRNA small nuclear RNAs tRNA transfer RNA
