# Supplementary material for: Identification of 15 candidate structured noncoding RNA motifs in fungi by comparative genomics
Source: BMC Genomics. 2017 Oct 13;18:785. doi: 10.1186/s12864-017-4171-y (PMC5640933; doi:10.1186/s12864-017-4171-y)
Supplement: Supplementary file 1 — Discovered ncRNA Information. Properties of and species for each ncRNA discovered. (DOCX 24 kb) [file 12864_2017_4171_MOESM1_ESM.docx]

**Additional file 1: Table S1.** Additional details for each ncRNA class. Note: The species listed are from the database RefSeq Release 68 only.

| ncRNA Class | Properties | Species (strains) |
| --- | --- | --- |
| HDV ribozyme variants | HDV ribozyme variants are similar to known members of this self-cleaving ribozyme class, except for (i) a C-G base-pair typically represents the otherwise more general Y-R base-pair at the ribozyme cleavage site, (ii) the P4 stem is partially replaced by an E loop RNA motif, and (iii) an additional nucleotide (most commonly a C residue) is inserted between the P2 and P3 stems. | *Ajellomyces capsulatus*  *Ajellomyces dermatitidis*  *Arthroderma benhamiae*  *Arthroderma gypseum*  *Arthroderma otae*  *Aspergillus clavatus*  *Aspergillus flavus*  *Aspergillus fumigatus*  *Aspergillus nidulans*  *Aspergillus niger*  *Aspergillus oryzae*  *Aspergillus terreus*  *Chaetomium globosum*  *Coccidioides posadasii*  *Gibberella zeae*  *Mycosphaerella graminicola*  *Nectria haematococca*  *Neosartorya fischeri*  *Paracoccidioides brasiliensis*  *Penicillium chrysogenum*  *Penicillium marneffei*  *Talaromyces stipitatus*  *Thielavia terrestris*  *Trichophyton rubrum*  *Trichophyton verrucosum*  *Uncinocarpus reesii* |
| SDC motif | This motif typically forms a small hairpin with an 11-base-pair stem. Each representative is located immediately upstream of a SAM (*S*-adenosylmethionine) decarboxylase (SDC) gene | *Ajellomyces dermatitidis*  *Aspergillus clavatus*  *Aspergillus fumigatus*  *Aspergillus nidulans*  *Aspergillus niger*  *Aspergillus oryzae*  *Aspergillus terreus*  *Chaetomium globosum*  *Neosartorya fischeri*  *Neurospora crassa*  *Paracoccidioides brasiliensis*  *Penicillium chrysogenum*  *Penicillium marneffei*  *Phaeosphaeria nodorum*  *Podospora anserine*  *Sclerotinia sclerotiorum*  *Uncinocarpus reesii* |
| *amd* motif | The motif likely adopts an elongated two-stem junction wherein the most-highly conserved nucleotides reside in the internal bulge and nucleotides in both P1 and P2 nearest to this bulge. Moreover, the nucleotides immediately upstream of P1, along with several others extending into the internal bulge, appear to code for a short uORF. In more than nine species, the associated gene is annotated as coding for an amidase enzyme. | Ajellomyces capsulatus;  Ajellomyces dermatitidis;  Aspergillus clavatus;  *Aspergillus nidulans*  *Aspergillus niger*  *Aspergillus oryzae*  *Aspergillus terreus*  *Botryotinia fuckeliana*  *Chaetomium globosum*  *Coccidioides immitis*  *Gibberella zeae*  *Magnaporthe oryzae*  *Myceliophthora thermophile*  *Nectria haematococca*  *Neosartorya fischeri*  *Neurospora crassa*  *Paracoccidioides brasiliensis*  *Penicillium chrysogenum*  *Penicillium marneffei*  *Phaeosphaeria nodorum*  *Podospora anserine*  *Talaromyces stipitatus*  *Uncinocarpus reesii* |
| *ies6* motif | This RNA motif forms an extended hairpin structure with one small and one large internal bulge. The hairpin loop conforms to a GNRA tetraloop sequence, which is frequently found in structured RNAs. The polarity of the RNA motif appears to be opposite to that of the mRNA, and therefore we speculated that this motif is likely present in an antisense RNA produced from the same genomic location as the *ise6* gene. | *Ajellomyces capsulatus*  *Ajellomyces dermatitidis*  *Arthroderma benhamiae*  *Arthroderma gypseum*  *Arthroderma otae*  *Aspergillus clavatus*  *Aspergillus nidulans*  *Aspergillus niger*  *Aspergillus oryzae*  *Aspergillus terreus*  *Chaetomium globosum*  *Coccidioides immitis*  *Gibberella zeae*  *Leptosphaeria maculans*  *Magnaporthe oryzae*  *Myceliophthora thermophile*  *Nectria haematococca*  *Neosartorya fischeri*  *Neurospora crassa*  *Paracoccidioides brasiliensis*  *Penicillium chrysogenum*  *Penicillium marneffei*  *Phaeosphaeria nodorum*  *Podospora anserine*  *Sclerotinia sclerotiorum*  *Sordaria macrospora*  *Talaromyces stipitatus*  *Thielavia terrestris*  *Trichophyton rubrum*  *Trichophyton verrucosum*  *Uncinocarpus reesii* |
| *hexA* motif | The predicted secondary structure includes a long hairpin interrupted by two small internal bulges. The motif is found in the putative 5´ UTRs or introns of genes encoding either hypothetical proteins (40%) or Woronin body protein Hex subunits (60%). | *Ajellomyces dermatitidis*  *Arthroderma otae*  *Aspergillus fumigatus*  *Aspergillus nidulans*  *Aspergillus niger*  *Aspergillus oryzae*  *Aspergillus terreus*  *Botryotinia fuckeliana*  *Coccidioides posadasii*  *Neosartorya fischeri*  *Paracoccidioides brasiliensis*  *Penicillium chrysogenum*  *Penicillium marneffei*  *Phaeosphaeria nodorum*  *Sclerotinia sclerotiorum*  *Talaromyces stipitatus*  *Trichophyton verrucosum*  *Uncinocarpus reesii* |
| SART-1 motif | The secondary structure of this motif has the potential to form at least two hairpins, although only P1 is supported by extensive evidence of covariation and the frequent presence of a UNCG tetraloop element. Nearly all representatives are located in the 5´ UTR of a gene similar to the mammalian *SART-1* (squamous cell carcinoma antigen recognized by T cells) gene. | *Ajellomyces capsulatus*  *Ajellomyces dermatitidis*  *Aspergillus clavatus*  *Aspergillus fumigatus*  *Aspergillus nidulans*  *Aspergillus niger*  *Aspergillus terreus*  *Coccidioides immitis*  *Coccidioides posadasii*  *Neosartorya fischeri*  *Paracoccidioides brasiliensis*  *Uncinocarpus reesii* |
| AU-rich hairpin motif | Only ten examples of the AU-rich hairpin motif were identified in fungal species. | *Rhizoctonia solani* |
| Atypical snoRNA motif | These motifs carry two regions that closely approximate the C box (AUGAUGY) and D box (CUGA). However, the apparent Cˊ box (AUGAGAC) and Dˊ box (CAGA) consensus sequences correspond to the consensus snoRNA sequences more poorly. | *Ajellomyces capsulatus*  *Ajellomyces dermatitidis*  *Aspergillus clavatus*  *Aspergillus flavus*  *Aspergillus nidulans*  *Aspergillus niger*  *Aspergillus terreus*  *Botryotinia fuckeliana*  *Candida albicans*  *Candida dubliniensis*  *Candida glabrata*  *Candida tropicalis*  *Chaetomium globosum*  *Clavispora lusitaniae*  *Coccidioides immitis*  *Coprinopsis cinerea*  *Cryptococcus gattii*  *Cryptococcus neoformans*  *Debaryomyces hansenii*  *Eremothecium cymbalariae*  *Gibberella zeae*  *Kluyveromyces lactis*  *Kluyveromyces thermotolerans*  *Laccaria bicolor*  *Leptosphaeria maculans*  *Lodderomyces elongisporus*  *Magnaporthe oryzae*  *Meyerozyma guilliermondii*  *Moniliophthora perniciosa*  *Myceliophthora thermophile*  *Mycosphaerella graminicola*  *Nectria haematococca*  *Neosartorya fischeri*  *Neurospora crassa*  *Paracoccidioides brasiliensis*  *Penicillium chrysogenum*  *Penicillium marneffei*  *Phaeosphaeria nodorum*  *Pichia pastoris*  *Podospora anserine*  *Postia placenta*  *Pyrenophora teres*  *Scheffersomyces stipites*  *Schizophyllum commune*  *Schizosaccharomyces japonicas*  *Schizosaccharomyces pombe*  *Sclerotinia sclerotiorum*  *Talaromyces stipitatus*  *Tetrapisispora phaffii*  *Thielavia terrestris*  *Trichophyton verrucosum*  *Uncinocarpus reesii*  *Vanderwaltozyma polyspora*  *Yarrowia lipolytica*  *Zygosaccharomyces rouxii* |
| Group I ribozymes | Over 200 examples of what appear to be previously unannotated group I self-splicing ribozymes were identified. | *Ajellomyces dermatitidis*  *Allomyces macrogynus*  *Aspergillus clavatus*  *Barnettozyma californica*  *Beauveria bassiana*  *Blastocladiella emersonii*  *Botryotinia fuckeliana*  *Brettanomyces custersianus*  *Candida albicans*  *Candida alimentaria*  *Candida chauliodes*  *Candida corydalis*  *Candida deformans*  *Candida frijolesensis*  *Candida galli*  *Candida jiufengensis*  *Candida labiduridarum*  *Candida maltose*  *Candida metapsilosis*  *Candida neerlandica*  *Candida norvegica*  *Candida orthopsilosis*  *Candida parapsilosis*  *Candida pseudojiufengensis*  *Candida sake*  *Candida salmanticensis*  *Candida theae*  *Candida zemplinina*  *Cantharellus cibarius*  *Ceratocystis cacaofunesta*  *Chaetomium globosum*  *Chaetomium thermophilum*  *Coccidioides immitis*  *Coccidioides posadasii*  *Coprinopsis cinerea*  *Cordyceps bassiana*  *Cordyceps brongniartii*  *Cyberlindnera jadinii*  *Cyberlindnera suaveolens*  *Debaryomyces hansenii*  *Dekkera bruxellensis*  *Epidermophyton floccosum*  *Flammulina velutipes*  *Fusarium circinatum*  *Fusarium graminearum*  *Fusarium solani*  *Ganoderma lucidum*  *Gibberella moniliformis*  *Gigaspora rosea*  *Glomus cerebriforme*  *Glomus intraradices*  *Glomus irregulare*  *Hyaloraphidium curvatum*  *Hypocrea jecorina*  *Kluyveromyces lactis*  *Lachancea kluyveri*  *Lentinula edodes*  *Leptosphaeria maculans*  *Madurella mycetomatis*  *Magnusiomyces magnusii*  *Marssonina brunnea*  *Meyerozyma guilliermondii*  *Millerozyma farinose*  *Moniliophthora roreri*  *Mortierella verticillata*  *Neosartorya fischeri*  *Ogataea thermophile*  *Peltigera malacea*  *Peltigera membranacea*  *Penicillium marneffei*  *Phakopsora meibomiae*  *Phakopsora pachyrhizi*  *Phlebia radiate*  *Pichia kluyveri*  *Pichia pastoris*  *Pleurotus ostreatus*  *Podospora anserine*  *Puccinia graminis*  *Pyrenophora teres*  *Rhizoctonia solani*  *Rhizopus oryzae*  *Saccharomyces paradoxus*  *Saccharomyces pastorianus*  *Sclerotinia sclerotiorum*  *Smittium culisetae*  *Sordaria macrospora*  *Talaromyces marneffei*  *Talaromyces stipitatus*  *Tilletia walker*  *Trametes cingulate*  *Tuber melanosporum*  *Ustilago maydis*  *Wickerhamomyces mucosus*  *Yarrowia lipolytica* |
| *rps0* motif | The *rps0* motif consists of two hairpins, one of which carries two conserved 5´-GGGGAAAG sequence elements partly located on each side of an internal bulge. Given the apparent symmetry of the *rps0* RNA sequence and its location adjacent to the *rpo0* gene, it seems possible that the motif might bind two or more RPS0 proteins to regulate expression of this ribosomal protein factor. | *Ajellomyces capsulatus*  *Ajellomyces dermatitidis*  *Arthroderma benhamiae*  *Arthroderma gypseum*  *Arthroderma otae*  *Aspergillus clavatus*  *Aspergillus flavus*  *Aspergillus fumigatus*  *Aspergillus nidulans*  *Aspergillus niger*  *Aspergillus terreus*  *Botryotinia fuckeliana*  *Chaetomium globosum*  *Coccidioides immitis*  *Coccidioides posadasii*  *Gibberella zeae*  *Leptosphaeria maculans*  *Myceliophthora thermophile*  *Mycosphaerella graminicola*  *Neosartorya fischeri*  *Neurospora crassa*  *Paracoccidioides brasiliensis*  *Penicillium chrysogenum*  *Penicillium marneffei*  *Phaeosphaeria nodorum*  *Podospora anserine*  *Pyrenophora teres*  *Sclerotinia sclerotiorum*  *Talaromyces stipitatus*  *Thielavia terrestris*  *Trichophyton rubrum*  *Trichophyton verrucosum*  *Uncinocarpus reesii* |
| *rps2* motif | All examples are located in the 5ʹ UTR of the gene encoding 40S ribosomal protein S2. Although there are few examples, there are four predicted base-pairs distributed between P1 and P2 that covary in a manner consistent with the predicted secondary structure. | *Candida albicans*  *Candida dubliniensis*  *Candida glabrata*  *Candida orthopsilosis*  *Lodderomyces elongisporus*  *Meyerozyma guilliermondii*  *Pichia pastoris*  *Scheffersomyces stipites* |
| *rps20* motif | All the representatives are located at the 3ʹ UTR of the 40S ribosomal protein S20. | *Eremothecium cymbalariae*  *Kazachstania Africana*  *Kluyveromyces lactis*  *Kluyveromyces thermotolerans*  *Naumovozyma castellii*  *Naumovozyma dairenensis*  *Tetrapisispora blattae*  *Tetrapisispora phaffii*  *Torulaspora delbrueckii*  *Vanderwaltozyma polyspora*  *Zygosaccharomyces rouxii* |
| *rpl7-L8-S3* motif | Representatives are located mostly in the introns of genes encoding 60S ribosomal protein L7 and L8, as well as the 40S ribosomal protein S3. | *Ajellomyces capsulatus*  *Ajellomyces dermatitidis*  *Arthroderma gypseum*  *Arthroderma otae*  *Aspergillus clavatus*  *Aspergillus flavus*  *Aspergillus fumigatus*  *Aspergillus nidulans*  *Aspergillus niger*  *Aspergillus terreus*  *Botryotinia fuckeliana*  *Chaetomium globosum*  *Coccidioides immitis*  *Gibberella zeae*  *Neosartorya fischeri*  *Neurospora crassa*  *Paracoccidioides brasiliensis*  *Penicillium marneffei*  *Phaeosphaeria nodorum*  *Podospora anserine*  *Sclerotinia sclerotiorum*  *Talaromyces stipitatus*  *Trichophyton rubrum*  *Uncinocarpus reesii* |
| *rpl7* motif | This is a small motif of approximately 30 nucleotides containing some conserved nucleotides in and around a central bulge. All examples of this RNA motif are located in the introns of 60S ribosomal protein L7 mRNAs. | *Ajellomyces capsulatus*  *Ajellomyces dermatitidis*  *Arthroderma benhamiae*  *Arthroderma otae*  *Aspergillus clavatus*  *Aspergillus fumigatus*  *Aspergillus nidulans*  *Aspergillus niger*  *Aspergillus oryzae*  *Aspergillus terreus*  *Coccidioides immitis*  *Coccidioides posadasii*  *Neosartorya fischeri*  *Paracoccidioides brasiliensis*  *Penicillium marneffei*  *Talaromyces stipitatus* |
| *rpl30* motif | This motif is comprised of a long 5ˊ region with little evidence for structure formation, followed by a region that appears to form a large hairpin structure with a well-conserved purine-rich internal loop. Most representatives are located in the 5´ UTR of genes encoding 60S ribosomal protein subunit L30. | *Ajellomyces capsulatus*  *Ajellomyces dermatitidis*  *Arthroderma benhamiae*  *Arthroderma gypseum*  *Arthroderma otae*  *Aspergillus clavatus*  *Aspergillus flavus*  *Aspergillus fumigatus*  *Aspergillus nidulans*  *Aspergillus niger*  *Aspergillus terreus*  *Botryotinia fuckeliana*  *Chaetomium globosum*  *Coccidioides immitis*  *Coccidioides posadasii*  *Gibberella zeae*  *Leptosphaeria maculans*  *Magnaporthe oryzae*  *Myceliophthora thermophile*  *Mycosphaerella graminicola*  *Nectria haematococca*  *Neosartorya fischeri*  *Neurospora crassa*  *Paracoccidioides brasiliensis*  *Penicillium chrysogenum*  *Penicillium marneffei*  *Phaeosphaeria nodorum*  *Podospora anserine*  *Pyrenophora teres*  *Sclerotinia sclerotiorum*  *Sordaria macrospora*  *Talaromyces stipitatus*  *Thielavia terrestris*  *Trichophyton rubrum*  *Trichophyton verrucosum*  *Uncinocarpus reesii* |
