# Supplementary material for: Identification of 15 candidate structured noncoding RNA motifs in fungi by comparative genomics
Source: BMC Genomics. 2017 Oct 13;18:785. doi: 10.1186/s12864-017-4171-y (PMC5640933; doi:10.1186/s12864-017-4171-y)
Supplement: Supplementary file 17 — Genome database lists. A listing of the genomic database files used for computational searches. (DOCX 13 kb) [file 12864_2017_4171_MOESM17_ESM.docx]

**Additional file 17: Table S2.** Genome database lists. The genome numbers are not continuous.

| fungi.1.1.genomic.fna | fungi.12.1.genomic.fna |
| --- | --- |
| fungi.2.1.genomic.fna | fungi.13.1.genomic.fna |
| fungi.3.1.genomic.fna | fungi.14.1.genomic.fna |
| fungi.4.1.genomic.fna | fungi.15.1.genomic.fna |
| fungi.5.1.genomic.fna | fungi.16.1.genomic.fna |
| fungi.6.1.genomic.fna | fungi.28.1.genomic.fna |
| fungi.7.1.genomic.fna | fungi.29.1.genomic.fna |
| fungi.8.1.genomic.fna | fungi.30.1.genomic.fna |
| fungi.9.1.genomic.fna | fungi.31.1.genomic.fna |
| fungi.10.1.genomic.fna | fungi.32.1.genomic.fna |
| fungi.11.1.genomic.fna |  |

Note: Fungal genomes were downloaded from NCBI <ftp://ftp.ncbi.nlm.nih.gov/refseq/release/fungi/>. The release number is RefSeq Release 68 (downloaded on 1/5/2015).
