# Supplementary material for: Identification of 15 candidate structured noncoding RNA motifs in fungi by comparative genomics
Source: BMC Genomics. 2017 Oct 13;18:785. doi: 10.1186/s12864-017-4171-y (PMC5640933; doi:10.1186/s12864-017-4171-y)
Supplement: Supplementary file 18 — PCR primers. DNA sequences of PCR primers used for the study. (DOCX 17 kb) [file 12864_2017_4171_MOESM18_ESM.docx]

**Additional file 18: Table S3.** PCR primers used for this study.

Primers for *rpl7* analysis were chosen to bind sequences in the exons flanking the intron of interest.

| Primers for RT-PCR | | | |
| --- | --- | --- | --- |
| Primers name | Sequence | RT-PCR products to be detected (nt) | Annotation |
| *rpl7*-F | 5´ CACCGTCCCTACTCAGGACC | Precursor: 437; Spliced: 253 | In each RT-PCR, use the reverse primer (for example RPL7-R) for the first strand DNA (cDNA) synthesis |
| *rpl7*-R | 5´ CTTGGGCTCCTCAGGAACGTAG |  |  |
| snoRNA-F | 5´ GTGTCGATTCAGAGTCGGAAGTC | Precursor: 702; Spliced: 469 |  |
| snoRNA-R | 5´ GATATGGCATAGGCGTGCACG |  |  |
| *ies6*-anti-F | 5´ TCTGAGTTCGGTTTCTCTCC | Antisense transcript：145 |  |
| *ies6*-anti-R | 5´ AAGTCCCAACTACGTACTCG |  |  |
| *ies6*-sense-F | 5´ CAAACCAAACTCCAGAAGCTCAAG | Sense transcript：623 |  |
| *ies6*-sense-R | 5´ GAGCACGGTGTGAGCACCTC |  |  |
| Primers for cloning | | | |
| *sdc*-F | 5´ GATCGAATTCCCTCCTCTTGAGCCTTTCAA | Use these two primers to infuse the SDC motif and partial SDC ORF to the luciferase reporter gene. | |
| *sdc*-R | 5´ GATCTCTAGAGGAGTGCCCTCGGAAGG |  |  |
